# Occupational kneeling and radiographic tibiofemoral and patellofemoral osteoarthritis

**DOI:** 10.1186/1745-6673-4-19

**Published:** 2009-07-13

**Authors:** Søren Rytter, Niels Egund, Lilli Kirkeskov Jensen, Jens Peter Bonde

**Affiliations:** 1 Department of Orthopaedics, Hospital Unit West, Herning, Denmark; 2 Department of Radiology, Aarhus University Hospital, Denmark; 3 Department of Environmental and Occupational Medicine, Copenhagen University Hospital, Bispebjerg, Denmark

## Abstract

**Background:**

The objective of our study was to evaluate the association between occupational kneeling and compartment specific radiographic tibiofemoral (TF) and patellofemoral (PF) osteoarthritis (OA).

**Methods:**

Questionnaire data and bilateral knee radiographs were obtained in 134 male floor layers and 120 male graphic designers (referents). Weight-bearing radiographs in three views (postero-anterior, lateral and axial) were classified according to joint space narrowing. After the exclusion of subjects with reports of earlier knee injuries the odds ratio (OR) with 95% confidence intervals (CI) of TF and PF OA was computed among floor layers compared to graphic designers in three age groups (≤ 49; 50–59; ≥ 60 years). Using logistic regression, estimates were adjusted for body mass index and knee-straining sports. In addition, the association between trade seniority and TF OA was assessed in age-adjusted test for trend analyses.

**Results:**

The prevalence of TF OA was significantly higher among floor layers aged 50–59 years compared to graphic designers (OR = 3.6, 95% CI = 1.1–12.0) while non-significant estimates were found in the young and elderly age groups. Furthermore, the adjusted OR of TF OA increased with trade seniority among floor layers (test for trend, OR = 2.2, 95% CI = 1.0–5.1), but not among graphic designers (OR = 1.2, 95% CI = 0.4–3.5). There were no significant differences regarding PF OA between the two occupational groups.

**Conclusion:**

Results corroborate the existence of a causal relationship between occupational kneeling and radiographic TF OA and suggest a dose-response association with trade seniority. An association between kneeling and PF OA was however doubtful. Apparent discrepancies between findings in different age groups are most likely reflecting selection bias.

## Background

Knee osteoarthritis (OA) is a common chronic joint disorder and a major source of disability. Knee OA is related to age and several other factors such as gender, genetic predisposition, previous knee injuries, obesity and some sports activities [1]. Causal relations with certain occupations and some occupational work activities have also been described [2-9]. A resent review showed a significant association between kneeling and knee OA with odds ratios (OR) ranging from 2.2–6.9 [10]. However, there has been sparse information in the literature concerning the distribution of compartment specific knee OA in relation to occupational kneeling. Floor layers particularly are exposed to repetitive and prolonged periods of kneeling work and only few jobs have the same level of knee demands as workers in this profession. It has been depicted that floor layers on average spend half of their daily working time in kneeling work positions [11].

Work retention among Danish senior citizen has been a major topic in recent years due to low unemployment rates. Therefore, with the object of raising the labour force a new law was passed by the Danish Ministry of Employment in 2006 that raised the age limit at which employees could retire. As the prevalence of OA increases with age this could be a future problem regarding the progression of knee OA among an aging workforce, especially in the construction industry.

To improve the possibility of preventive intervention strategies regarding the development of occupationally related tibiofemoral (TF) and patellofemoral (PF) OA, it is important to identify possible risk factors. Thus, with the object of evaluating the relationship between kneeling work and knee OA we examined the prevalence of radiographic TF and PF OA and its compartmental distribution (medial and lateral) among floor layers highly exposed to kneeling work-strains compared to a group of low-level exposed graphic designers.

## Methods

### Study participants

A Danish sample of 286 male floor layers and 370 male graphic designers were established from trade union rosters comprising members aged 36–70 years in 2004. Workers were recruited in Copenhagen (capital city) and Aarhus (second largest city), Denmark. Graphic designers were included as reference group. They worked at visual display units and their work did not include knee-demands. Floor layers install linoleum, carpet and vinyl floorings, and their work tasks involve removal of old floorings, priming, grinding, filling, gluing, welding, and mounting skirting boards (plastic). The majority of Danish floor layers and graphic designers are members of a trade union and in Denmark these two trade groups are comparable regarding the level of education and socio-economic status.

A self-administered questionnaire was mailed to the initial study sample with a response rate of 88% and 78% among floor layers and graphic designers, respectively. Respondents with reports of previous acute knee injuries defined as fractures involving the knee joint, meniscus lesions or cruciate ligament ruptures were excluded from the study, leaving an eligible sample of 231 floor layers and 258 graphic designers. Written informed consent to perform a radiographic examination was obtained from 134 floor layers (Copenhagen n = 88; Aarhus n = 46) and 120 graphic designers, all from Copenhagen (Table 1). One participant contributed only with one PF joint (unilateral patelloectomy).

**Table 1:** Study and eligible sample for the radiographic study, stratified in age groups

	**Floor layers**					**Graphic designers**				
	
	Study sample	Survey respondents	Knee injury	Eligible sample*	Study participants	Study sample	Survey respondents	Knee injury	Eligible sample*	Study participants
**Age group**	N	n (%)^†^	n (%)^‡^	n (%)	n (%)^§^	N	n (%)^†^	n (%)^‡^	n (%)	n (%)^§^
**≤ 49 years**	115	99 (86)	11 (11)	88 (77)	43 (49)	40	33 (83)	4 (12)	29 (73)	7 (24)
**50–59 years**	111	99 (89)	4 (4)	95 (86)	72 (76)	155	128 (83)	13 (10)	115 (74)	73 (64)
**≥ 60 years**	60	55 (92)	7 (13)	48 (80)	19 (40)	175	129 (74)	15 (12)	114 (65)	40 (35)

**Total**	286	253 (89)	22 (9)	231 (81)	134 (58)	370	290 (78)	32 (11)	258 (70)	120 (47)

* Respondents with previous knee injuries excluded

^† ^% of study sample

^‡ ^% of survey respondents

^§ ^% of eligible sample

Permission from the Central Danish Region Committee on Biomedical Research Ethics was obtained before commencement of the investigation.

### Questionnaires

The questionnaire addressed information about employment and trade seniority, history of knee complaints, knee injuries (fractures, menisci, cruciate ligament or muscle injuries) and knee-straining sports experience defined as ever participated in: football, handball, badminton, tennis, volleyball, ice hockey or weight lifting. Knee complaints were defined as ache, pain, or nuisance during the past 12 months. Questions about musculoskeletal complaints were consistent with the Nordic Musculoskeletal Questionnaire [12].

### Radiographs

Radiographs of both knees were obtained in the standing and almost one leg weight-bearing position with the knee in 20–30 degree flexion in three views: postero-anterior (PA), lateral and axial of the PF joint space. A standardized examination technique with a device supporting the knee allowed adjustment for optimal visualization of the medial and lateral TF and PF joint spaces without fluoroscopy [13,14]. Antero-posterior (AP) radiographs of the pelvis and hips were also conducted in all participants.

### Radiographic scoring and grading

Radiographs were read and scored on workstations with 2 K screens by one experienced musculoskeletal radiologist (NE). The reader was blinded to any medical history of knee disorders among participants. Due to differences in the appearance of radiographic images among radiographs obtained in Copenhagen and Aarhus, blinding of occupational affiliation was incomplete regarding participants from Aarhus (n = 46) who were all floor layers. Blinding of occupational affiliation was complete concerning all participants from Copenhagen (n = 208).

Radiographic scoring comprised assessment of the medial and lateral joint spaces of both the TF and PF compartment using a modified Ahlbäck scale (grade 0–6) of joint space narrowing (JSN) and subchondral bone attrition [15]. The following grades were defined: grade 0 = normal; grade 1 = minimal but definite JSN (25% JSN); grade 2 = moderate JSN (50% JSN); grade 3 = severe JSN (75% JSN); grade 4 = obliteration of the joint space, "bone on bone but no attrition"; grade 5 = < 5 mm attrition of subchondral bone and grade 6 = ≥ 5 mm bone attrition. Developed from previous studies and routine diagnostics, a set of specific criteria's illustrated by an atlas of standard radiographs were used to avoid readers drift. The main criteria for the assessment of JSN, grade 1 were based on a comparison between the same joint spaces of the normal contralateral knee in each participant. When both TF joint spaces were affected a minimal joint space of 4 mm were used [16]. According to this classification we defined OA as JSN ≥ grade 1 in at least one knee joint space and patterns of involvement into medial or lateral TF OA and PF OA. In addition, the presence and size of osteophytes were registered, but these findings were not used in our classification of OA. Radiographs of the hips were classified as normal or abnormal (JSN or alterations due to other pathology).

### Reliability of radiographic scoring

The intra-reader reliability was tested in respect to the separation between a normal joint space and a minimal but definite JSN (≥ grade 1) as well as the scoring of different grades of JSN. All participants scored with knee OA (n = 61) were randomly mixed by an independent IT-technologist in a file of digital images, with the knees of 26 participants scored as normal (n = 193). The same reader randomly and blindly re-scored these radiographs (n = 87). Among 87 participants (173 knees; one patelloectomy), which were read twice 6 medial TF, 4 medial PF and 2 lateral PF joint spaces had their grading changed; eight from grade 0 to 1, and four from grade 1 to 0. None of the reassessed joint spaces changed more than one grade and no changes were observed regarding the lateral TF joint spaces. The intra-reader agreement was 96.6% for the assessment of the TF compartment, and 96.5% for the PF compartment.

### Data analyses and statistics

The study sample was divided into three age strata (≤ 49, 50–59, ≥ 60 years). In each stratum we computed the OR with 95% confidence intervals (CI) of radiographic TF and PF OA among floor layers compared to graphic designers. Using logistic regression, models were adjusted for body mass index (BMI; < 25, 25–29, ≥ 30 kg/m^2^) and knee-straining sports experience (yes/no). In additional analyses we computed the association between trade seniority and TF OA in age-adjusted test for trend analyses and examined the compartmental distribution of medial and lateral TF and PF OA. The relationship between hip alterations as a possible explanation of referred knee pain was examined among participants with reports of knee complaints, but without concomitant radiographic signs of knee OA.

Statistical analyses were performed using Stata (version 8.0, StataCorp LP, College Station, TX, USA).

## Results

### Characteristics of study participants

Participation in the study varied considerably, by age. Attendance was highest among participants aged 50–59 years whereas those younger than 50 years and older than 60 years were underrepresented, especially among graphic designers (Table 1). Graphic designers were older and had higher trade seniorities compared to floor layers. The proportion of lifetime participation in any knee-straining sports was slightly higher among graphic designers than floor layers, but in respect to BMI the two groups were comparable (Table 2).

**Table 2:** Characteristics of study participants; floor layers (n = 134) and graphic designers (n = 120)

	**Floor layers**		**Graphic designers**	
**Age **(years) *mean, range*	52.6	39–68	57.9	40–70
Trade seniority* (years) *mean, range*	29.2	3–49	35.6	8–54
**BMI**^† ^(kg/m^2^) *mean, range*	26.4	20–41	26.0	17–42
**Knee-straining sports**^‡ ^*n, (%)*	71	(53)	80	(67)
**Knee osteoarthritis**^§ ^*n, (%)*				
***Grade 1***				
- Tibiofemoral	10	(8)	2	(2)
- Patellofemoral	5	(4)	8	(7)
***Grade 2–3***				
- Tibiofemoral	7	(5)	7	(6)
- Patellofemoral	4	(3)	8	(8)
***Grade 4–6***				
- Tibiofemoral	5	(4)	6	(5)
- Patellofemoral	3	(2)	5	(4)

* Years of employment in the trade

^† ^Body mass index

^‡ ^Defined as football, handball, badminton, tennis, volleyball, ice hockey, and weight lifting

^§ ^Combined tibiofemoral and patellofemoral osteoarthritis; floor layers n = 3, graphic designers n = 6

Twenty-four percent (n = 61) of participants were classified as having radiographic knee OA, 33 with unilateral and 28 with bilateral OA. According to the worst affected knee and compartment there was a diverse distribution between the two occupations. Nineteen (14.2%) floor layers and 9 (7.5%) graphic designers were classified as having isolated TF OA while isolated PF OA was found among 9 (6.7%) floor layers and 15 (12.5%) graphic designers, respectively. Combined OA in both the TF and PF compartments was found in 3 (2.2%) floor layers and 6 (5.0%) graphic designers (Table 2). There were no significant differences in the distribution of OA between the right and left knee either within or between occupational groups. Osteophytes were present in all knees with OA (≥ grade 1), while no osteophytes were registered in knees with normal joint spaces except for one knee in a floor layer.

Knee complaints were common among subjects with OA (Table 3). Additionally, workers with knee complaints participated more often in the study than workers without and this selective participation was much more pronounced among graphic designers than among floor layers in the young and the old age strata (Table 4).

**Table 3:** Proportion of knee complaints among floor layers and graphic designers with tibiofemoral (TF) or patellofemoral (PF) osteoarthritis (OA)

	**Floor layers**						**Graphic designers**					
	
	TF OA			PF OA			TF OA			PF OA		
**Age groups**	N*	n^†^	(%)	N*	n^†^	(%)	N*	n^†^	(%)	N*	n^†^	(%)
**≤ 49 years**	5	3	(60)	2	2	(100)	1	1	(100)	2	1	(50)
**50–59 years**	12	9	(75)	9	7	(78)	4	4	(100)	7	3	(43)
**≥ 60 years**	5	2	(40)	1	0	-	10	7	(70)	12	8	(67)

* Attendees in the age group with TF or PF OA

^† ^Knee complaints during the past 12 months

**Table 4:** Proportion of knee complaints among floor layers and graphic designers from the study sample, stratified into age groups

	**Floor layers**						**Graphic designers**					
	
	Study attendance*		Study non-attendance^†^				Study attendance*		Study non-attendance^†^			
	
**Age groups**	N^‡^	n^§ ^(%)	N^‡^	n^§ ^(%)	RR	95% CI	N^‡^	n^§ ^(%)	N^‡^	n^§ ^(%)	RR	95% CI
**≤ 49 years**	43	23 (53)	45	19 (42)	1.3	0.8–2.0	7	4 (57)	22	5 (23)	2.5	0.9–6.9
**50–59 years**	72	41 (57)	23	9 (39)	1.5	0.8–2.5	73	23 (32)	42	8 (19)	1.7	0.8–3.4
**≥ 60 years**	19	6 (32)	29	12 (41)	0.8	0.4–1.7	40	19 (48)	74	12 (16)	2.9	1.6–5.4

Risk ratio (RR) with 95% confidence interval (CI) among those who agreed to attend the radiographic study compared to those who declined

* Floor layers (n = 134); graphic designers (n = 120)

^† ^Floor layers (n = 97); graphic designers (n = 138)

^‡ ^Total in each age group

^§ ^Knee complaints during the past 12 months

### Radiographic knee osteoarthritis

Floor layers had a higher prevalence of TF OA compared to graphic designers. After adjustment, floor layers aged 50–59 years had a 3.6 times greater likelihood (OR = 3.6, 95% CI = 1.1–12.0) of having TF OA than graphic designers at the same age. Yet, a significant association was only found among floor layers in this age group (Table 5). Table 6 illustrates the distribution of OA by lateral and medial TF and PF joint space involvement. The medial TF compartment was affected mostly in both trades. However, the prevalence of medial TF OA was twice as high among floor layers (11.1%) aged 50–59 years compared to graphic designers (5.5%). In this age stratum (50–59 year), lateral TF OA was only observed among floor layers (5.6%).

**Table 5:** Radiographic knee osteoarthritis (OA) according to the worst affected knee and compartment among floor layers (n = 134) compared to graphic designers (n = 120)

	**Floor layers**		**Graphic designers**			
	
	Total	OA	Total	OA		
	N	n (%)	N	n (%)	OR*	95% CI
**≤ 49 years**	43		7			
Tibiofemoral		5 (12)		1 (14)	1.1	0.1–13.1
Patellofemoral		2 (5)		2 (29)	0.1	0.01–1.3
**50–59 years**	72		73			
Tibiofemoral		12 (17)		4 (6)	3.6	1.1–12.0
Patellofemoral		9 (13)		7 (10)	1.3	0.5–3.8
**≥ 60 years**	19		40			
Tibiofemoral		5 (26)		10 (25)	1.9	0.4–7.8
Patellofemoral		1 (5)		12 (30)	0.1	0.01–1.1

Odds ratio (OR) with 95% confidence interval (CI)

* Adjusted for body mass index and knee-straining sports activities

**Table 6:** Proportion of medial and lateral tibiofemoral or patellofemoral osteoarthritis (OA) relative to the worst affected knee and compartment among floor layers (n = 134) and graphic designers (n = 120)

	**Floor layers**			**Graphic designers**		
	
	Total	Medial	Lateral	Total	Medial	Lateral
	N	n (%)	n (%)	N	n (%)	n (%)
**≤ 49 years**	43			7		
Tibiofemoral OA		4 (9.3)	1 (2.3)		1 (14.3)	0 -
Patellofemoral OA		1 (2.3)	1 (2.3)		0 -	2 (28.6)
**50–59 years**	72			73		
Tibiofemoral OA		8 (11.1)	4 (5.6)		4 (5.5)	0 -
Patellofemoral OA		6 (8.3)	3 (4.2)		3 (4.1)	4 (5.5)
**≥ 60 years**	19			40		
Tibiofemoral OA		5 (26.3)	0 -		7 (17.5)	3 (7.5)
Patellofemoral OA		1 (5.3)	0 -		7 (17.5)	5 (12.5)

The prevalence of PF OA was only slightly higher among floor layers aged 50–59 years compared to graphic designers (OR = 1.3, 95% CI = 0.5–3.8), and the distribution between medial and lateral PF OA showed only minor differences in this age stratum. The prevalence of PF OA were oppositely higher among graphic designers in the youngest (OR = 0.1, 95% CI = 0.01–1.3) and oldest age strata (OR = 0.1, 95% CI = 0.01–1.1).

Changing the cut-off for radiographic scoring of knee OA from grade 1 to grade 2 revealed the same trend among floor layers compared to graphic designers in the age group 50–59 years, although the difference did not reach statistical significance. The OR of TF OA was 3.0 (95% CI = 0.8–12.0) and PF OA 1.6 (95% CI = 0.4–6.0).

Restricting analyses to floor layers (n = 88) and graphic designers (n = 120) from Copenhagen (all blinded in regard to occupational affiliation to the reader) did not alter the observed trend as the OR for TF OA in the age group 50–59 years was 3.6 (95% CI = 1.0–13.0) and for PF OA 1.9 (95% CI = 0.6–5.7).

The association between trade seniority and TF OA within floor layers and graphic designers are illustrated in Table 7. Age-adjusted test for trend analyses due to an incremental increase in the risk of TF OA at each level of trade seniority, revealed a higher OR among floor layers (OR = 2.2, 95% CI = 1.0–5.1) compared to graphic designers (OR = 1.2, 95% CI = 0.4–3.5). Comparing floor layers to graphic designers the adjusted OR was 2.8 (95% CI = 0.4–21.7) and 3.5 (95% CI = 1.3–9.7) in the stratum with 21–30 and ≥ 31 years of trade seniority, respectively.

**Table 7:** Tibiofemoral (TF) osteoarthritis (OA) among floor layers (n = 134) and graphic designers (n = 120) relative to trade seniority

	**Floor layers**				**Graphic designers**			
	
	Total	TF OA			Total	TF OA		
**Seniority**	N	n (%)	OR*	95% CI	N	n (%)	OR*	95% CI
**≤ 20 years**	29	2 (6.9)	1.0	-	8	1 (12.5)	1.0	-
**21–30 years**	44	6 (13.6)	2.3	0.4–12.8	26	2 (7.7)	1.4	0.1–21.4
**≥ 31 years**	61	14 (23.0)	5.0	0.9–28.5	86	12 (14.0)	1.6	0.1–17.5

Odds ratio (OR) with 95% confidence interval (CI)

* Adjusted for age, body mass index and knee-straining sports activities

Test for trend due to incremental increase in the risk of TF OA at each seniority level: floor layers OR* = 2.2, 95%

CI = 1.0–5.1; graphic designers OR* = 1.2, 95% CI = 0.4–3.5

Radiographic hip alterations were recorded among 6 (4.5%) floor layers and 12 (10.0%) graphic designers. Among those, 5 floor layers and 8 graphic designers had isolated hip alterations without concomitant radiographic signs of knee OA. Having hip alterations, the likelihood of enduring knee complaints were raised among both floor layers and graphic designers. The adjusted OR was 1.9 (95% CI = 0.3–12.6) among floor layers, and 2.6 (95% CI = 0.6–12.1) among graphic designers.

## Discussion

We observed a higher prevalence of radiographic TF OA among floor layers aged 50–59 years, but not in the younger and elder age groups. These apparently conflicting findings are most likely explained by selection bias. First, participation rates differed strongly between age groups and were particularly low among young and elderly in the reference group. Second, the proportion of workers with knee complaints that participated in the study was considerably higher among graphic designers than among floor layers, especially in the youngest and oldest age strata. Third, results revealed a high proportion of knee complaints among subjects with TF OA irrespectively of trade affiliation. Accordingly, participants with knee complaints (which is correlated with knee OA) were over-represented among graphic designers compared to floor layers. It is therefore most likely that risk estimates in the young and elderly age strata have been biased towards low risk values while findings in the 50–59 years age stratum, where a high participation rate was obtained, are unbiased. Furthermore, differential selection of workers towards different occupations depending on their health status may be inventible in occupations with high physical demands, and a healthy-worker selection may also have influenced results either in terms of primary selection of more healthy workers into the trade or in terms of longer survival in the trade of more healthy workers [17]. However, such selection mechanisms would typically result in an underestimation of the investigated association. Considering these aspects the interpretation of our results support the hypothesis of a causal relation between occupational kneeling and TF OA.

An earlier study among Danish floor layers found an increased prevalence of TF OA among floor layers ≥ 50 years compared to carpenters and compositors [8]. However, radiographs were conducted with the subjects lying supine. This may have lowered the power of the study as it is recognized that weight-bearing radiographs provide a more accurate assessment of JSN compared to non-weight-bearing examinations [18]. In a Finnish study, Kivimäki *et al *compared carpet and floor layers with painters [9]. They found a significant association between osteophytosis of the knees and occupation, but no differences in relation to JSN. Participants included in this study were relatively young (25–49 year) and therefore provides limited power to detect work-related effects due to the low prevalence of OA in this age range. A recent register-based cohort-study by Järvholm *et al *showed a significant increased relative risk of surgically treated knee OA among Swedish floor layers compared to white-collar workers [7]. These studies among others, support our findings of an association between kneeling work activities and knee OA.

Knowledge about mechanisms concerning the development of occupational knee OA has been sparse. However, it has been argued that OA is initiated when healthy cartilage is exposed to traumatic or chronic conditions that shift loads to regions of cartilage that are not conditioned to chronic repetitive loading [19]. Direct and repetitive loading of the knee joint could possibly induce micro-injuries with structural breakdown of collagen and result in OA. Alternatively, repetitive loading might increase the risk of meniscal or ligamentous injuries, which could cause malalignment of knee dynamics [20,21]. Studies have shown that loss of soft-tissue stability alter loading patterns and may cause progression of degenerative changes [19,22]. Nagura *et al *showed that TF joint forces increased considerable during deep knee flexion, especially forces acting on the posterior part of the tibia plateau [23]. During knee flexion, TF contact surfaces are displaced posteriorly, and between 90–110 degree knee flexion contact areas are decreased to 60% [24]. Given that contact forces increases and the contact area decrease during deep knee flexion this could be a contributing factor in the formation of degenerative changes and explain the higher prevalence of TF OA among floor layers exposed to repetitive and prolonged periods of kneeling. Additionally, the risk of TF OA increased with trade seniority among floor layers, but not among graphic designers. This could support the hypothesis that accumulated knee-strains increases the risk of TF OA.

The medial TF compartment was most often affected in both trades, which are in accordance with the "normal" distribution of TF OA [15]. However, the prevalence of medial TF OA was twice as high among floor layers aged 50–59 than among graphic designers. During knee flexion medial contact forces is larger than forces acting in the lateral compartment and the medial TF compartments absorb approximately 70% of the total load passing through the knee joint. This load imbalance between compartments is created by an adduction moment in the knee during ambulation [25]. Imbalance of loads across the knee joint could explain the different distribution of medial and lateral TF OA observed between floor layers and graphic designers. The distribution of medial and lateral PF OA was almost even in both occupations, but results cannot be compared with the literature since a distinction between compartment specific PF JSN rarely have been made in previous studies [16].

Compared to TF OA, there seemed to be a weaker, if any, association between knee-straining work and PF OA. Earlier reports concerning risk factors associated with PF OA have been conflicting and sporadic [26,27]. Cooper *et al *[20] found a positive although non-significant association between occupational kneeling and PF OA and the same pattern have been found among Asians with non-occupational floor activities [28]. PF contact forces are the resultant of the quadriceps tendon force and the patellar tendon force. PF contact forces gradually increase during knee flexion, but only up to 80-degree flexion [29]. Opposed to the TF compartment, PF contact forces decrease and the PF contact area increase above 80-degree flexion angels [25]. The majority of kneeling work tasks among floor layers are performed in knee angles above 90-degree flexion. During such work procedures most of the direct related stress between the knee and underlay are located around the tibiae tubercle and not between the patella and underlay. These different biomechanical characteristics of the TF and PF compartments may theoretically influence the pathogenetic mechanism involved in the development of OA, and could explain a lacking association between kneeling work demands and PF OA.

Our analyses indicated that radiographic hip alterations raised the probability of having concomitant knee complaints among attendees without knee OA. Knee pain referred from pathology of the hip must therefore be kept in mind among subjects with unexplained knee complaints [30,31]. Floor layers are also exposed to heavy lifting and a causal relation between heavy lifting; hip and knee OA have been argued [32]. Radiographs of the hips were only conducted in the AP view in our study. Still, analyses revealed only very few cases with hip JSN and our results did not indicate an association between kneeling, heavy lifting, and hip OA.

To ensure consistent radio-anatomical appearance of the knee joint we used routine imaging techniques in the assessment of the TF and PF joint spaces. Radiographs were obtained in the standing and almost one leg weight-bearing position with the knee in 20–30 degree flexion. This represent a modified technique introduced by Ahlbäck [15] and has been applied to assess knee OA in previous studies [33-35]. We used the same radiographic technique added by a devise, which supported the knee in all three views. This technique allows adjustment in the positioning of the knee to obtain the intended appearances of the joint spaces in the PA and axial views, guided by the lateral view [14]. The technique was therefore comparable to those using fluoroscopy [36,37].

The Kellgren-Lawrence (KL) grading system of OA has the advantage of a global assessment of OA in joints and the scale of degenerative joint deterioration (grade 0 – 4) has been widely adopted in the rheumatologic literature [38]. With the aid of the "Atlas of individual radiographic features in osteoarthritis" this scoring method has obtained a high reliability [39-41]. However, using the KL grading system appears less appropriate in our material where the objective was to compare specific features of OA in each of the four joint spaces between the two study groups [42]. We therefore used a grading system that encompassed all stages of OA from early JSN to bone attrition as proposed by Ahlbäck and in addition measured the presence and size of osteophytes [15]. Our assessment of minimal but definite JSN (grade 1) was mainly based on a semi-quantitative and quantitative comparison between the same joint spaces of the right and left knee [14]. Using these criteria's a high intra-reader agreement was achieved. In addition, sensitivity analyses changing the threshold of OA from grade 1 to grade 2 did not alter results as floor layers aged 50–59 years still had a higher prevalence of OA compared to graphic designers and furthermore, exclusion of floor layers from Aarhus who was not blinded in regard to occupational affiliation to the reader did not modify results. The relevance of using our grade 1 of JSN in the classification of OA may be confirmed by the high intra-reader agreement and in particular by the concomitant presence of osteophytes in all knees with OA (≥ grade 1) and the lack of osteophytes in knees, except one, with joint spaces assessed as normal. Osteophytes may represent a reparative process in posttraumatic and non-degenerative conditions [42-44]. However, Boegård *et al *reported a high correlation between marginal osteophytes at radiography and MR detected cartilage defects in both the TF and PF compartments [44,45].

## Conclusion

Unlike earlier studies this study illustrates not only the distribution of TF and PF OA, but also the distribution between the medial and lateral compartments. Our results suggest that occupational kneeling pose a risk in the development of medial TF OA, and furthermore that there seems to be a dose-response association between trade seniority and TF OA among floor layers. There were on the other hand no association between kneeling work and PF OA. However, the power of the study is limited due to low participation rate and there will be a need to corroborate or refuse current findings in additional studies.

## Competing interests

The authors declare that they have no competing interests.

## Authors' contributions

SR participated in the design of the study, in the acquisition of data, performed the statistical analyses, and participated in the interpretation of data. NE participated in the design of the study, assessed all radiographs, and participated in the analyses and the interpretation of data. LKJ and JPB participated in the design of the study and in the analyses and the interpretation of data. All authors have been involved in drafting the manuscript and approved the final version of the manuscript.
